# Nerve Growth Factor Is Responsible for Exercise-Induced Recovery of Septohippocampal Cholinergic Structure and Function

**DOI:** 10.3389/fnins.2018.00773

**Published:** 2018-11-01

**Authors:** Joseph M. Hall, Fernando Gomez-Pinilla, Lisa M. Savage

**Affiliations:** ^1^Behavioral Neuroscience Program, Department of Psychology, Binghamton University, Binghamton, NY, United States; ^2^Department of Integrative Biology and Physiology, University of California, Los Angeles, Los Angeles, CA, United States

**Keywords:** NGF, BDNF, acetylcholine, exercise, basal forebrain, hippocampus, nestin

## Abstract

Exercise has been shown to improve or rescue cognitive functioning in both humans and rodents, and the augmented actions of neurotrophins within the hippocampus and associated regions play a significant role in the improved neural plasticity. The septohippocampal circuit is modified by exercise. Beyond an enhancement of spatial working memory and a rescue of hippocampal activity-dependent acetylcholine (ACh) efflux, the re-emergence of the cholinergic/nestin neuronal phenotype within the medial septum/diagonal band (MS/dB) is observed following exercise (Hall and Savage, 2016). To determine which neurotrophin, brain-derived neurotrophic factor (BDNF) or nerve growth factor (NGF), is critical for exercise-induced cholinergic improvements, control and amnestic rats had either NGF or BDNF sequestered by TrkA-IgG or TrkB-IgG coated microbeads placed within the dorsal hippocampus. Hippocampal ACh release within the hippocampus during spontaneous alternation was measured and MS/dB cholinergic neuronal phenotypes were assessed. Sequestering NGF, but not BDNF, abolished the exercise-induced recovery of spatial working memory and ACh efflux. Furthermore, the re-emergence of the cholinergic/nestin neuronal phenotype within the MS/dB following exercise was also selectively dependent on the actions of NGF. Thus, exercise-induced enhancement of NGF within the septohippocampal pathway represents a key avenue for aiding failing septo-hippocampal functioning and therefore has significant potential for the recovery of memory and cognition in several neurological disorders.

## Introduction

Exercise improves an array of health outcomes, including the enhancement of learning and memory, particularly under pathological conditions. Upregulation of neurotrophins is a crucial mechanism by which exercise affects cognitive function (Neeper et al., 1996; Cotman et al., 2007), and each neurotrophin has a unique role in improving cognitive outcome. Brain-derived neurotrophic factor (BDNF) is considered to play an essential role in mediating the pro-cognitive effects of exercise via the enhancement of hippocampal neurogenesis, dendritic complexity, and synaptic plasticity (Vaynman et al., 2004; Stranahan et al., 2007; Wrann et al., 2013; Vivar and van Praag, 2017). Nerve growth factor (NGF) is essential for normal development as well as the functioning of mature cholinergic neurons in the basal forebrain. Across the lifespan, NGF is retrogradely transported from the hippocampus to MS/dB cholinergic neurons where it influences structure (both soma size and dendritic complexity) and modulates the activities of choline acetyltransferase and acetylcholinesterase (Conner et al., 2009; Isaev et al., 2017). The loss of NGF leads cholinergic neuronal atrophy (Koh et al., 1989; Sofroniew et al., 1990). In preclinical models of neurological disease, NGF treatment reverses the effects of lesions and age-related degeneration of basal forebrain cholinergic neurons, including the recovery of learning and memory (Tuszynski and Blesch, 2004).

Although the expression of NGF is amplified in the MS/dB and hippocampus following exercise (Neeper et al., 1996; Chae and Kim, 2009; Hall and Savage, 2016), its unique role in brain plasticity following exercise has not been determined. This is surprising given that NGF is critical for maintaining and promoting cholinergic neuron survival and specifically upregulates ChAT+ phenotypic expression in the basal forebrain (Fischer et al., 1987; Gustilo et al., 1999; Tuszynski et al., 2015), and that forebrain cholinergic neurons are imperative for learning and memory (Conner et al., 2009; Easton et al., 2012; Ballinger et al., 2016).

Exercise increases muscarinic receptor density and high affinity choline uptake in the hippocampus (Fordyce and Farrar, 1991) and increases the number of neurons expressing choline acetyltransferase (ChAT+) in the horizontal diagonal band (Ang et al., 2006). One month of voluntary wheel running also increased the afferent input from medial septum to newly born hippocampal neurons (Vivar et al., 2016). Furthermore, we demonstrated (Hall and Savage, 2016) that voluntary wheel running recovered both spatial working memory and behaviorally stimulated hippocampal ACh efflux, as well as rescued degenerating MS/dB cholinergic neurons in an animal model of alcohol-related amnestic syndrome, or Korsakoff Syndrome. Interestingly, the restorative effect of exercise was primarily seen in the unique phenotype of ChAT+ neurons that also express nestin. About 30% of the forebrain ChAT+ neurons express nestin and these mature neurons are more responsive to stimulation (Gu et al., 2002; Hendrickson et al., 2011; Zhu et al., 2011). We hypothesized that the ChAT/nestin neuronal phenotype in the MS/dB are a malleable type of cholinergic neuron that is very responsive to neurotrophin modulation and are influential in driving activity-dependent hippocampal ACh release and associated behaviors (Hall and Savage, 2016).

However, given that both BDNF and NGF have durable trophic effects, such as rescuing atrophied forebrain ChAT+ neurons (Morse et al., 1993; Tuszynski and Gage, 1995), it is unclear which neurotrophin is instrumental for the exercise-dependent rescue of the septohippocampal cholinergic system. Exercise has been shown to increase both BDNF and NGF levels in the hippocampus in normal rats (Neeper et al., 1996), and exercise recovers BDNF and NGF neurotrophin deficits in rats made amnestic by thiamine deficiency (Hall and Savage, 2016). Our laboratory employs the pyrithiamine-induced thiamine deficiency (PTD) model of Korsakoff Syndrome, the alcohol-related amnestic disorder, to study system level interactions in neuropathology, neurochemical dysfunction and behavioral impairment. Beyond the traditional thalamic pathology associated with thiamine deficiency, there are also reductions in cortical and hippocampal behaviorally activated acetylcholine (ACh) efflux that has been linked to the loss of forebrain cholinergic populations (see Savage et al., 2012).

In the present study, we blocked the actions of either BDNF or NGF with microbeads coated with either TrkB-IgG or TrkA-IgG antibodies (Vaynman et al., 2004; Griesbach et al., 2009), during the voluntary exercise bout. This sequestering technique has been effectively used to block neurotrophin action for weeks throughout the hippocampus (Vaynman et al., 2003, 2004, 2006; Miladi-Gorji et al., 2011). To determine the functional output of voluntary exercise, with and without sequestered neurotrophins, spontaneous alternation performance, activity-dependent ACh efflux and unbiased stereological assessment of MS/dB cholinergic neuronal phenotypes were assessed at a critical time point after exercise (see Hall et al., 2014; Hall and Savage, 2016).

The data revealed that the nestin cholinergic neuronal phenotype was the most responsive to both pathology and exercise. Furthermore, sequestering the actions of NGF, throughout exercise, blocked both the functional and structural cholinergic improvements advanced by exercise in amnestic rats. In control rats, sequestering NGF and BDNF lead to small but significant decreases in spatial alternation, but only inhibiting the actions of NGF suppressed activity-dependent ACh efflux after exercise. The nestin cholinergic phenotype in the intact brain also responded to the sequestering of NGF. Such results suggest that exercise modulates the cholinergic forebrain through an NGF-dependent mechanism and the nestin cholinergic phenotype is exceptionally reactive to NGF levels.

## Materials and Methods

### Subjects

For the behavioral assessment, ACh efflux measures and cell counting procedures, adult male Sprague-Dawley rats (*N* = 133), weighing between 275 and 300 g (Envigo, Indianapolis, IN, United States) were used throughout this experiment. The goal was to conclude with eight rats per group, so additional rats were included to account for attrition due to treatment or surgery. There was a low level of attrition, thus some groups contain more than eight rats (see below). Rats were placed in a temperature-controlled vivarium (20–22°C), and maintained on a 12-h light/dark cycle with light onset at 07:00 h. All procedures followed full accordance with the Institutional Animal Care and Use Committee of Binghamton University and the National Institute of Health: Guide for the Care and Use of Laboratory Animals (9th ed., National Academies Press, 2014). Additionally, these rats were all pair-housed, all had standard bedding in clear plastic cages and had access to an enrichment wood chew block for the entire duration of the study.

A separate cohort (*N* = 42) of adult male Sprague-Dawley rats (275–300 g, Envigo, Indianapolis, IN, United States) were used to initially determine whether delivery of unilateral or bilateral TrkA-IgG and TrkB-IgG coated microbeads abolished the exercise-induced increase in neurotrophin protein levels, and whether this suppression persisted throughout exercise.

### Pyrithiamine-Induced Thiamine Deficiency (PTD)

**FIGURE 1:**
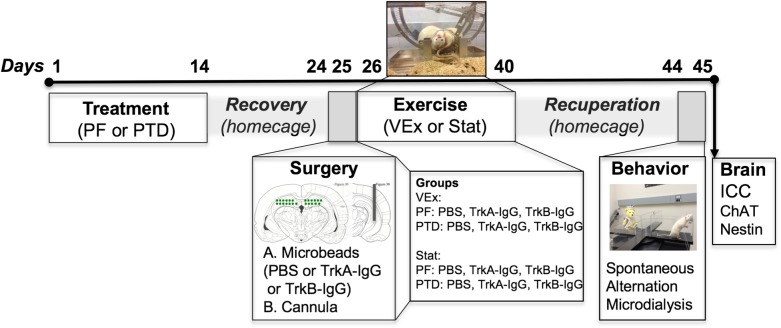
Experimental timeline indicating that the subjects were randomly split between PTD and PF treatment. After treatment recovery, rats received a dorsal hippocampal infusion of either TrkB-IgG-, TrkA-IgG- or saline-coated microbeads, in addition to a cannulation to measure ACh efflux. Immediately following surgery rats were split into two groups, either a VEx (voluntary exercise) group where rats received running wheels attached to a home-cage for 2-weeks, or a Stat group (stationary) that had exposure to immobile wheels for this 2-week duration. Following the exercise exposure, rats were placed back into normal homecages and were tested 2-weeks later on a spontaneous alternation task with microdialysis to collect hippocampal ACh dialysate samples, followed by perfusion and assessment of ChAT and nestin cellular morphology in the MS/dB. The images indicating microbead delivery and hippocampal cannulations were adapted from Paxinos and Watson (Paxinos and Watson, 2014).

The details of the standard Pair-fed (PF) and PTD treatment have been described extensively in our previously published studies (see Roland and Savage, 2009; Hall et al., 2014; Hall and Savage, 2016). Briefly, pyrithiamine hydrobromide injections (0.25 mg/kg; Sigma-Aldrich Corp., St. Louis, MO, United States) were given for 14–16 days in conjunction with *ad libitum* thiamine-deficient chow, until the appearance of severe neurological symptoms, at which rats were given a large bolus injection of thiamine. This standard treatment induces the neuropathology similar to KS. PF control rats received pyrithiamine hydrobromide equivalent to the amount consumed by PTD-treated rats in addition to thiamine hydrochloride (0.4 mg/kg; Sigma-Aldrich Corp., St. Louis, MO, United States) in order to replete thiamine levels. Following PTD and PF treatment all rats were placed back onto a normal diet consisting of Purina rat chow for a 10-day recovery period prior to surgery. An experimental overview and timeline of the study can be seen in Figure 1.

### Microbead Preparation

To scavenge available BDNF and NGF, during exercise, fluorescent microbeads (Green Retrobeads^TM^ IX; LumaFluor Inc., Durham, NC, United States) were treated with either recombinant human TrkB Chimeric Fusion Protein (Fc chimera; Cat: 688-TK-100, R&D Systems, Minneapolis, MN, United States) or reconstituted recombinant TrkA Fc chimera (Cat: 175-TK-050; R&D Systems) (see Vaynman et al., 2004, 2006; Ying et al., 2008). These microbeads slowly release the coated substances.

Fluorescent latex microbeads were added to a centrifuge tube containing 1.0 mL of 0.1% BSA (in PBS) at a ratio of 1:5, respectively (100 μL green fluorescent microbeads to 500 μL recombinant human TrkB/Fc or 100 μL green fluorescent microbeads to 1,000 μL recombinant human TrkB/Fc chimera). To prepare the PBS control microbeads, 1.0 mL of the 0.1% bovine serum albumin (BSA) solution (in PBS) was added to a centrifuge tube, and 100 μL of the green fluorescent microbeads were added in addition to 500 μL PBS (0.1M from above), at the 1:5 respective ratio. This control was shown to be effective by Ying et al. (2008). The solutions were mixed and stored at 4°C overnight. The next day, each solution was centrifuged for 30 min at 14,000 rpm. Supernatant was removed, and the pellet was re-suspended with 10 μL of sterile nanopure dH_2_O.

### Microbead Infusion and Cannulation Surgeries

Hippocampal cannulations (8 mm guide cannula; Synaptech Technology Inc., Marquette, MI, United States) were performed on all rats using the following coordinates from bregma [(AP) = -0.53 mm, (ML) = -0.51 mm, (DV) = -0.42 mm; see Savage et al., 2011; Hall and Savage, 2016]. Microbead solutions were delivered as previously described (see Vaynman et al., 2004, 2006). Pilot data indicated that blockade of NGF required TrkA-IgG coated microbeads to be delivered bilaterally to both hemispheres. To block BDNF, we added a bilateral TrkB-IgG group to address the issue of laterality with blocking NGF, despite that a unilateral dose of TrkB-IgG coated microbeads were sufficient at reducing both the contralateral and ipsilateral hemispheres. Infusions of TrkB-IgG or TrkA-IgG coated microbead solution, 2 μL, was inserted into a hemisphere, which sequesters BDNF or NGF for 14 days of exercise (see Ying et al., 2008). The respective microbead solution was delivered to the dorsal hippocampus [(AP) = -0.38 mm, (ML) = +0.26 mm, (DV) = -0.37 mm; relative to bregma] over a period of 15 min. Immediately following surgery rats were placed in pairs (two rats/cage) in the exercise running wheel chamber apparatuses for the remainder of the experiment, and the two rats per cage shared one running wheel (locked or unlocked, see below).

### Exercise Paradigm

Identical exercise parameters were adapted from our previous published studies (Hall et al., 2014; Hall and Savage, 2016). Microbead and cannulated PTD- and PF-treated rats were randomly assigned into one of two exercise conditions: (1) a voluntary exercise condition (VEx) in which a running wheel (Lafayette Instrument Company, Lafayette, IN, United States) was attached to the home cage or (2) a sedentary condition with a locked exercise wheel (Stat). Rats were pair-housed and food restricted to 16 g/rat/day. Food restriction has been shown to increase wheel running (see Sherwin, 1998; Lee et al., 2002). Furthermore, rats remained in the wheel running apparatus for a period of 2-weeks, with daily running (cumulative m/day) recorded via AWM^®^ software (Lafayette Instrument Company).

### BDNF and NGF Western Blot Expression

To determine the degree and persistence of the treated microbeads to suppress BDNF and NGF levels following exercise, non-treated rats were into the following conditions: Stat + PBS coated microbeads (*n* = 6), VEx + PBS coated microbeads (*n* = 6), VEx + unilateral TrkB-IgG coated microbeads (*n* = 6), VEx + bilateral TrkA-IgG (*n* = 6), VEx + unilateral TrkA-IgG (*n* = 6), VEx + bilateral TrkA-IgG coated microbeads (*n* = 6). These rats were sacrificed on day 14 of VEx.

Hippocampal tissue was dissected and each sample was diluted (1:10) and normalized to the same protein level (50 μg). Total protein was determined with a BCA protein assay (Thermo Fisher, cat# 23227, Waltham, MA, United States). Samples along with the standard ladder and manufacturer recommended positive controls were loaded into Novex^®^ pre-cast 16% polyacrylamide gels, and electrophoresed (125 V; 2.5 h). Western blots were chosen to measure extracellular NGF and BDNF protein levels since enzyme-linked immunosorbent assay (ELISA) analysis was not possible because the hippocampal tissue contained fluorescent latex microspheres that would directly interfere with BDNF quantification because the beads would fluoresce at the specific wavelengths. Western blots for BDNF and NGF were run on separate gels with the standard ladder, and were initially tested for their respective positive control (BDNF: glioblastoma human whole cell lysate, cat# U-87; NGF: mouse brain extract, cat# sc-2253; Santa Cruz Biotechnology, Santa Cruz, CA, United States). Blots were blocked for 60 min with 5% BSA in TBS+Tween. Next, blots were incubated into respective primary antibodies at a dilution of 1:500 in 5% BSA overnight at 4°C (BDNF: affinity purified rabbit polyclonal, cat# sc-546, Santa Cruz Biotechnology; NGF: rabbit polyclonal, cat# sc-548, Santa Cruz Biotechnology). The following day, blots were incubated for 60-min in secondary antibody (1:1000; bovine anti-rabbit IgG-HRP secondary antibody, cat# sc-2370, Santa Cruz Biotechnology), prior to development of film using Pierce ECL Western blotting detection reagents (Thermo Fisher Scientific). Hypoxanthine phosphoribosyltransferase-1 (HPRT-1; goat polyclonal, 1:1000, Santa Cruz Biotechnology) was used as a housekeeper, for within-subject normalization of BDNF/NGF per HPRT-1 values. Analysis of NGF/BDNF bands were performed using the program ImageJ (Gómez-Pinilla and Ying, 2010; Ding et al., 2011) to determine size of bands in comparison to the housekeeper HPRT-1.

### Behavioral *in vivo* Microdialysis and Maze Testing

Given that the Western blot data demonstrated equal effectiveness for unilateral and bilateral placement of TrkB-IgG, but not TrkA-IgG, in sequestering the corresponding neurotrophin, both the PF and PTD groups contained the following microbead conditions: (1) VEx TrkB-IgG (bilateral + unilateral; PF *n* = 15; PTD *n* = 15); (2) Stat TrkB-IgG (bilateral + unilateral; PF *n* = 14; PTD *n* = 14); (3) VEx TrkA-IgG (bilateral; PF *n* = 8; PTD *n* = 8), (4) Stat TrkA-IgG (bilateral; PF *n* = 8; PTD *n* = 9); (5) VEx + PBS (bilateral + unilateral; PF *n* = 11; PTD *n* = 12) and (6) Stat + PBS (bilateral + unilateral; PF *n* = 10; PTD *n* = 8). Bilateral PBS conditions were added as well as unilateral conditions to ensure delivery would not affect behavior or neurochemical analyses.

Rats were returned to standard pair-housing conditions after VEx/Stat for 9 days prior to being handled daily for an additional 5 days prior to behavioral testing as a 14-day period has been demonstrated to be needed to see exercise-induced changes in the forebrain cholinergic system (see Hall and Savage, 2016). Spontaneous alternation testing procedures, in conjunction with microdialysis collection of hippocampal ACh efflux, was conducted with procedures published in our previous work (see Anzalone et al., 2010; Hall and Savage, 2016; Fernandez and Savage, 2017). Spontaneous alternation is a spatial task that is sensitive to the cholinergic system as well as the functioning of the hippocampus and basal forebrain (for review, see Lalonde, 2002). Microdialysate samples were collected every 6-min during baseline (total: 18-min), during spontaneous alternation (total: 18-min), and post-baseline (total: 18-min). An alternation was defined as entry into four different arms in overlapping successive sequences of four arm entries (for example, in the successive arm entries of B, A, D, C, A, D, C, A, D, B, C, D, B, A; the first sequence of BADC was an alternation, but the next 4-arm sequence ADCA was not). The percent alternation score is equal to the ratio of actual alternations to possible alternations (total alternations/[trial number - 3] × 100).

### Tissue Preparation and Immunohistochemistry

Rats were transcardially perfused with 4% methanol-free formaldehyde in PB. Brains were extracted, kept in formalin for 24-h, and placed into 30% sucrose for 5 days prior to coronal sectioning (40 μM) on a freezing microtome (Lecia, Instruments, Wetzler, Germany). Slices from the HPC coordinates were mounted, coverslipped, and viewed under fluorescence to determine Trk(A/B)-IgG location. Furthermore, we examined cannula placement within the hippocampus on Nissl stained sections and sequential sections were used to identify cholinergic phenotypes (see below).

### Immunohistochemistry—ChAT/Nestin and Unbiased Stereological Cellular Quantification

**FIGURE 2:**
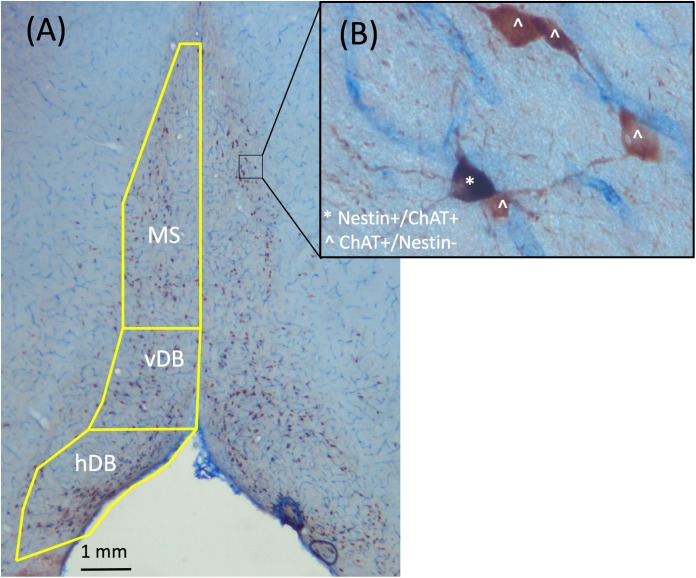
Representative images taken from the MS/dB with 2.5× **(A)** and 40× **(B)** objectives of ChAT+/Nestin+ dual brightfield immunohistochemical stain. ChAT+/Nestin– cells were stained red, while Nestin+/ChAT+ neurons were stained purple. Cells labeled with ˆ indicate they are ChAT+/Nestin– while cells labeled with ^∗^ indicate they are Nestin+/ChAT+.

Since the latex fluorescent microbeads retrogradely label neurons in the MS/dB, a brightfield stain was employed to allow for analysis the co-localization of Nestin with ChAT (see Figure 2). Thus, we developed a brightfield co-labeling stain to assess the Nestin/ChAT populations of neurons as well as the overlap in the Nestin and ChAT neuronal populations. Six sections from every subject (sampling every 5th section) spanning the MS/DB were treated with a standardized ICC protocol (see Roland and Savage, 2009; Hall and Savage, 2016; Fernandez and Savage, 2017). Following rinsing (PB), quench and blocking steps, sections were first processed with the ChAT primary antibody (1:200, goat polyclonal anti-ChAT, cat# AB144P; Millipore, Billerica, MA, United States), and where then incubated in a secondary antibody (biotinylated anti-goat IgG 1:100, cat# BA-5000; Vector Laboratories, Burlingame, CA, United States). This was followed a PB rinse and then incubation in an avidin-biotin complex (cat# PK-6100; Vector Laboratories). Finally, ChAT was developed in an ImmPACT NovaRED solution kit (cat# SK-4805; Vector Laboratories), which marks cholinergic neurons as reddish brown under a brightfield microscope. Next, the stained ChAT tissue was treated with the Nestin primary antibody (1:200; mouse monoclonal anti-Nestin, cat# MAB353; Millipore). The next day, tissue was rinsed (PB) and incubated into a pre-made secondary antibody solution (ImmPRESS AP anti-mouse IgG-alkaline phosphatase, cat# MP-5402, Vector Laboratories). Lastly, the tissue was rinsed and developed in a Vector blue substrate solution (cat# SK-5300, Vector Laboratories).

Unbiased stereological assessment is commonly employed to assess the cholinergic basal forebrain neuronal population (see Yeo et al., 1997; Gritti et al., 2006; Roland et al., 2014) and allows for a better assessment of neuronal quantification since this measure is unbiased and is currently the most validated and widely used method (see West et al., 1991; Naumann et al., 2002). We used unbiased stereology to estimate ChAT+ and Nestin+ cell populations in the MS/DB (see Yoder and Pang, 2005; Savage et al., 2007; Anzalone et al., 2010; Hall and Savage, 2016; Fernandez and Savage, 2017). Slides were coded to ensure that the experimenter that performed cell counts was blind to all experimental conditions. A Carl Zeiss Microscope (Zeiss Axioscope 2-Plus, Thornwood, NY, United States) with an attached digital camera (DVC-1310; DVC Company, Austin, TX, United States) containing a motorized stage was used in combination with Stereo Investigator software (MicroBrightField Bioscience, Williston, VT, United States) on a computer containing a Windows XP operating system. As depicted in Figure 2, for cholinergic (ChAT) staining ChAT+/Nestin- cells appear reddish while Nestin+/ChAT- cells appear bluish. However, since > 95% of Nestin+ neurons are also ChAT+ neurons (Nestin+/ChAT+), nearly all the Nestin+ neurons co-express ChAT+ and therefore are purple. Quantification of neuronal phenotypes were performed using a 40× dry-objective lens, and counting was performed using the optical fractionator function, as previously described (see Hall and Savage, 2016; Fernandez and Savage, 2017).

### High Performance Liquid Chromatography (HPLC)

Microdialysis samples were submitted to HPLC (HTEC-500, Eicom USA, San Diego, CA, United States) to assess ACh content (see Savage et al., 2011; Hall and Savage, 2016; Fernandez and Savage, 2017). The detection of the system is 5 fmol and sample fmol value calculations were performed via the software Envision^®^ (Eicom).

### Statistical Procedures and Data Availability

All data were expressed as means ± SEM. All data were analyzed using the statistical program SPSS Statistics for Macintosh (Version 21.0; IBM Corp., Armonk, NY, United States). Cumulative distance data was analyzed as a mixed model repeated measures ANOVA with the one between-subjects factors Treatment (PF vs. PTD) and one within-subjects factor Day (across 14 days). For spontaneous alternation, ChAT and Nestin populations, we employed a three-factor between-subjects ANOVA with Treatment (PF vs. PTD), Exercise (VEx vs. Stat) and Microbeads (TrkB-IgG, TrkA-IgG, PBS) as between-subjects variables. Hippocampal ACh efflux was analyzed as a mixed-model repeated measures ANOVA with Treatment, Exercise, and Microbeads as between subjects factors, with Sample Time (blocks 1–3) collapsed within Phase (baseline, maze, after) as two within-subjects factors. Furthermore, in cases of any significant main effects or interactions, we ran *post hoc* tests for type of Microbead (TrkB-IgG, TrkA-IgG, PBS) using Scheffe’s test. The raw numerical data supporting this manuscript will be made available by the authors, without undue hesitation, to any qualified researcher.

## Results

### Both Unilateral or Bilateral Hippocampal Infusions of TrkB-IgG Effectively Blocked Exercise-Induced Amplification of BDNF, Whereas Bilateral Infusion of TrkA-IgG Was More Effective Than Unilateral Infusion of TrkA-IgG at Blocking the Exercise-Induced Rise of NGF

**FIGURE 3:**
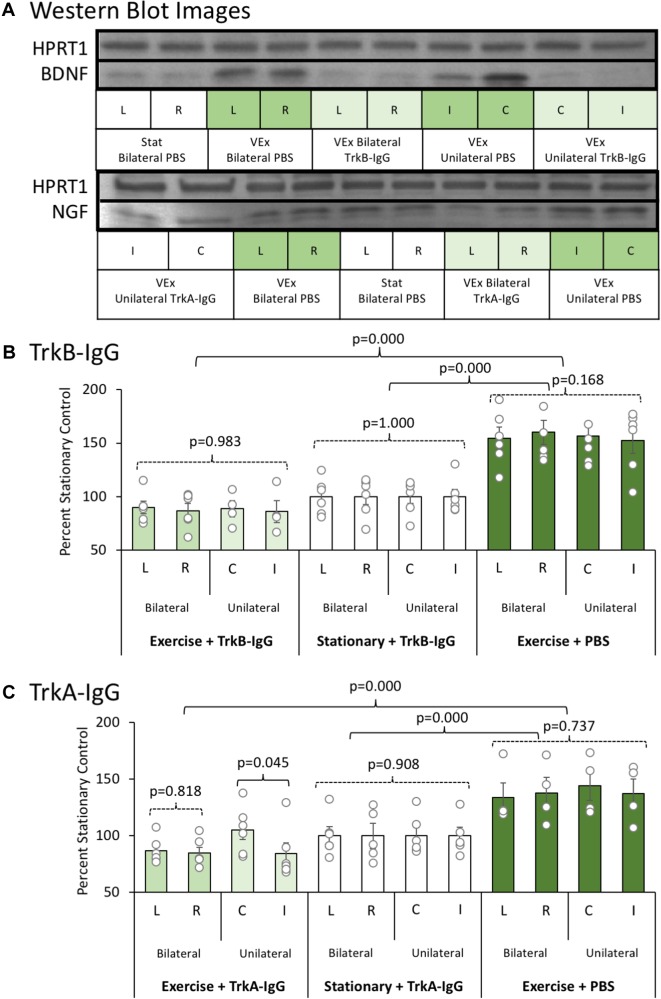
**(A)** Representative Western blot images for BDNF and NGF proteins as a function of exercise condition (VEx = Exercise; Stat = Stationary control wheels), substance coating the microbeads (PBS, TrkB-IgG; TrkA-IgG), and whether the beads were implanted on both sides of the dorsal hippocampus (bilateral) or only on one side (unilateral) in control rats. Hypoxanthine phosphoribosyltransferase 1 (HPRT1) was used as a housekeeper. **(B)** The mean ± SEM of percent control of BDNF with values normalized to HPRT1 levels. Relative to the stationary wheel condition (Stat), control rats that voluntarily exercised had persistent increased BDNF levels. However, both bilateral and unilateral hippocampal infusion of TrkB-IgG coated microspheres completely suppressed the exercise-induced increase in BDNF such that they were comparable to the BDNF level in sedentary rats. **(C)** The mean ± SEM of percent control of NGF with values normalized to HPRT1 levels. Relative to the stationary wheel condition (Stat), rats that voluntarily exercised had persistent increased NGF levels. However, unlike TrkB IgG, the infusion of TrkA IgG coated microspheres differentially suppressed exercise-induced increases in NGF as a function of whether the infusion was bilateral or unilateral within the hippocampus. Specifically, in rats that exercised, only bilateral infusion of TrkA-IgG coated microspheres led to a significant suppression of NGF, while the unilateral infusion of TrkA-IgG coated microspheres did not significantly decrease NGF levels in the hippocampus in the contralateral hemisphere.

An initial experiment was conducted to ensure that the microbead procedures (unilateral vs. bilateral hippocampal implantation of microbeads coated with either TrkA-IgG or TrkB-IgG) could inhibit available NGF and BDNF for an extended time period. Representative blots for NGF and BDNF are shown in Figure 3A, along with the housekeeper HPRT1 bands. Whereas bilateral hippocampal implantation of TrkA-IgG microbeads was more effective at reducing available NGF than unilateral implantation of TrkA-IgG microbeads [*F*(1,10) = 4.54, *p* = 0.045, see Figure 3C], this was not the case for of the implantation of TrkB-IgG microbeads, as unilateral and bilateral implantation did not differ in reducing available BDNF [*F*(1,8) = 0.058, *p* = 0.816], similar to previous studies indicating that injection of unilateral TrkB-IgG microbeads are sufficient to reduce BDNF levels in the HPC (see Vaynman et al., 2004; Griesbach et al., 2009). As shown in Figure 3B, both unilateral and bilateral hippocampal implantation of TrkB-IgG microbeads were both similarly effective at reducing available BDNF [*F*(1,30) = 140.210, *p* = 0.000; see also Vaynman et al., 2006]. Since injections of TrkB-IgG delivered unilaterally and bilaterally led to a similar blockade of BDNF, the two TrkB-IgG conditions were combined. To demonstrate the selectivity of the microbead procedures, levels of the alternative growth factor were also measured. The TrkB-IgG treated microbeads selectively blocked the exercise-induced increase in BDNF, but did not sequester NGF, which significantly rose (about 30%) with exercise [*F*(1,30) = 14.87, *p* = 0.001]. Furthermore, the TrkA-IgG treated microspheres, while blocking the exercise-induced rise in NGF, did not inhibit the exercise-induced rise in BDNF [*F*(1,30) = 27.31, *p* = 0.000]. Importantly, we observed no differences between the unilateral and bilateral groups on behavior, ACh efflux or cell counts, were detected as a function of unilateral or bilateral implantation of TrkB-microbeads (all *p*’s > 0.201). However, given that the unilateral condition suppressed NGF less than the bilateral condition, only the bilateral Trk-A condition was used for behavioral, ACh and cellular analyses.

### PTD-Treated Rats Run Less Than PF-Treated Control Rats and Microbead Infusion Does Not Impact Wheel Running

**FIGURE 4:**
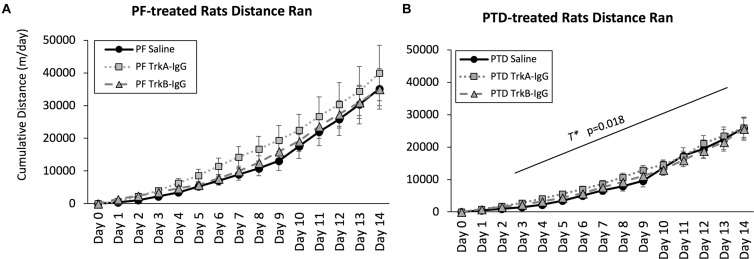
Cumulative distance ran throughout the 2-weeks of wheel running for PF rats **(A)** and PTD rats **(B)**. Distance traveled is expressed as meters per day ± SEM. PTD rats ran less than control PF rats from days 3 to 14. There were no differences observed in running as a function of blocking BDNF (TrkB-IgG) or NGF (TrkA-IgG). T^∗^ indicates a significant effect of Treatment.

Because sphericity was violated for the within-subjects factor of Day [χ^2^(90) = 3241.33, *p* = 0.0001], a Greenhouse–Geisser correction was applied for the analyses. As shown in Figure 4, a main effect of Treatment indicated that PF-treated control rats ran more compared with PTD-treated rats [*F*(1,63) = 6.37, *p* = 0.014]. Analysis at each day indicated that PTD-treated rats ran less than PF-treated rats from days 3 to 14 [all *F*’s(1,69) < 6.00, *p* < 0.02]. Similar to our previous data (see Hall et al., 2014; Hall and Savage, 2016), we observed the greatest difference toward the end of exercise exposure as indicated by a Treatment × Day interaction [*F*(1.102,69.43) = 5.60, *p* = 0.018]. Importantly, there was no effect of the microbeads on the cumulative distance that rats ran [*F*(2,63) = 0.70, *p* = 0.50].

### The Exercise-Induced Enhancement of Spontaneous Alternation Behavior in PTD-Rats Was Selectively Blocked by Sequestering of NGF, but Not BDNF

**FIGURE 5:**
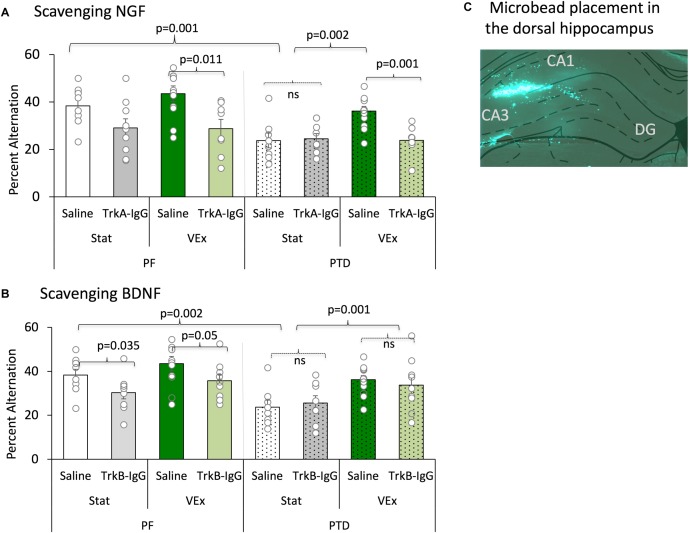
Spontaneous alternation is displayed with the sequestering of NGF [**(A)** (TrkA-IgG) and BDNF **(B)** (TrkB-IgG)], and is expressed as mean percent alternation ± SEM. Exercise (green bars) completely ameliorated the spontaneous alternation impairment in PTD-treated rats; conversely, blocking NGF (light green bars) completely abolished the exercise related recovery in PTD rats, and diminished performance in PF rats. Blocking BDNF impaired performance in PF control rats, regardless of exercise status. In contrast, inhibiting the actions of BDNF had no effect on the behavior of PTD-treated rats. Microbead placement is observed from a green fluorescent image taken of the dorsal hippocampus with 5× objective lens **(C)**.

As previously reported, Figure 5 depicts that PTD-treatment impairs spontaneous performance [*F*(1,121) = 16.25, *p* = 0.0001], but exercise did recover this deficit [*F*(1,121) = 8.253, *p* = 0.005]. Although exercise did not enhance spontaneous alternation performance in PBS-microbead treated PF rats, [*F*(1,19) = 1.471, *p* = 0.240], the effect of exercise was significant in PBS treated PTD rats [*F*(1,18) = 12.953, *p* = 0.002]. Furthermore, exercise fully recovered the behavioral impairment in PTD-treated rats: PTD rats exposed to VEx with PBS-treated microbeads did not differ from PF rats [*F*(1,20) = 0.464, *p* = 0.504].

Importantly, the sequestering neurotrophins with microbeads was critical for the exercise-induced recovery of spontaneous alternation performance [main effect of microbeads: *F*(1,121) = 8.541, *p* = 0.005], and follow-up analyses revealed that the action of NGF were necessary to for exercise to recover alternation behavior in PTD-treated rats (Figure 5A). As depicted, PTD-treated rats with TrkA-IgG microbeads, to inhibit the action of NGF, did not show any improvement in alternation performance as a function of exercise [*F*(1,15) = 0.49, *p* = 0.828]. Additionally, in PF VEx rats the TrkA-IgG microbeads caused a significant impairment in alternation performance [*F*(1,17) = 8.264, *p* = 0.011], compared with PF VEx rats that were administered PBS-treated microbeads. No significant effect of TrkA-IgG was seen in either PF rats [*F*(1,17) = 4.204, *p* = 0.06] or PTD rats [*F*(1,15) = 0.044, *p* = 0.837] without exercise.

In contrast, the TrkB-IgG microbeads did not block the actions of exercise (see Figure 5B) in the PTD group. The spontaneous alternation performance of PTD VEx rats that received TrkB-IgG microbeads did not differ from PTD VEx rats with PBS-treated microbeads [*F*(1,25) = 0.856, *p* = 0.364]. Additionally, the TrkB-IgG microbeads had no effect in PTD Stat rats in comparison to PTD stat rats with PBS-treated microbeads [*F*(1,20) = 0.209, *p* = 0.652].

Although exercise did not improve alternation performance in PF rats, the sequestration of BDNF decreased spontaneous alternation performance selectively in PF rats regardless of exercise condition. PF VEx rats treated with TrkB-IgG microbeads did have a reduced spontaneous alternation performance relative to PF VEx rats with PBS-coated microbeads [*F*(1,24) = 4.28, *p* = 0.05]. Even the alternation scores of stationary PF rats were affected sequestering the neurotrophins: Blocking the actions of BDNF in sedentary PF rats decreased alternation behavior [*F*(1,24) = 5.053, *p* = 0.035]. Thus, it appears that control and amnestic rats have differential sensitivity to blocking the actions of BDNF and NGF, with BDNF appearing critical for normal spatial working memory performance in control rats, while NGF appears critical for the exercise-induced rescue of spatial working memory in PTD-treated rats.

### Arm Entries During Spontaneous Alternation Were Not Affected by PTD Treatment, Exercise or Microbeads Treatment

Overall, we did not observe any effect of PTD Treatment, Exercise, or Microbeads manipulation on the number of arms entered during spontaneous alternation testing [Treatment: *F*(1,121) = 1.76, *p* = 0.187, Exercise: *F*(1,121) = 0.002, *p* = 0.963, Microbeads: *F*(1,121) = 1.502, *p* = 3.14; Means ± SEM: PBS Microbeads: PF-VEx: 29.90 ± 3.15, PF-Stat: 35.60 ± 3.29, PTD-VEx: 28.42 ± 2.50, PTD-Stat: 36.38 ± 6.13; TrkA-IgG Microbeads: PF-VEx: 37.63 ± 5.68, PF-Stat: 25.00 ± 2.91, PTD-VEx: 22.88 ± 2.42, PTD-Stat: 26.11 ± 3.96; TrkB-IgG Microbeads: PF-VEx: 32.67 ± 3.66, PF-Stat: 32.14 ± 3.46, PTD-VEx: 33.07 ± 3.50, PTD-Stat: 28.71 ± 3.00]. Furthermore, there were no interactions between the variables on arm entries.

### Basal Levels of Hippocampal ACh Are Reduced by the Sequestration of NGF in PTD Rats

**FIGURE 6:**
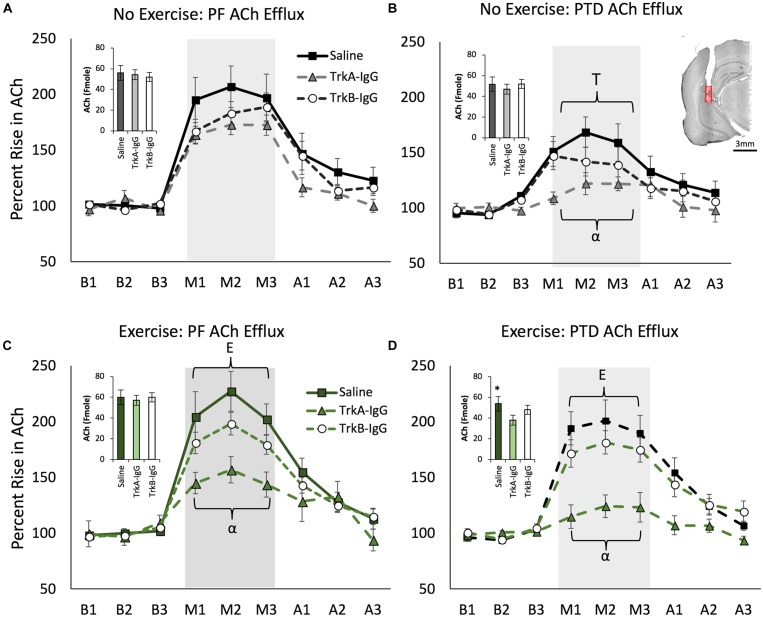
Hippocampal acetylcholine (ACh) efflux measured throughout baseline (B1–B3), spontaneous alternation (M1–M3) and post-baseline (A1–A3) phases. Inset graphs represent basal ACh levels in femtomoles. PTD-treatment reduced ACh efflux during maze testing **(A)** vs. **(B)**. In the no exercise condition (Stat), sequestering NGF with TrkA IgG reduced ACh efflux during maze testing in PTD rats **(B)**. Exercise increased ACh efflux in both PF control and PTD-rats **(C,D)**. In PTD VEx rats **(D)**, exercise rescued the impaired ACh levels to the level of control rats. However, sequestering NGF suppressed behaviorally activated ACh efflux from exercise in both PF and PTD rats **(C,D)**. **E** indicates a significant effect of exercise; **T** indicates a significant effect of treatment, ^∗^ indicates a significant effect of TrkA-IgG microbeads on basal ACh and **α** indicates a significant effect of blocking NGF. Within **(B)** is an illustrative example hippocampal placement of the microdialysis cannula. The red region represents the probe membrane.

A main effect of PTD treatment indicated that there was a reduction in basal levels of ACh compared with PF control rats [*F*(1,121) = 7.40, *p* = 0.01], while exercise did not affect basal ACh levels [*F*(1,121) = 3.14, *p* = 0.079]. As shown in Figure 6 (insets), basal hippocampal ACh levels were reduced by microbead delivery [*F*(2,121) = 12.06, *p* = 0.01], and Scheffe’s *post hoc* test indicated that these levels were significantly reduced by approximately 15% with the sequestration of NGF, via TrkA-IgG coated microbeads, in the PTD VEx group (*p* = 0.01), but not via blockade of the actions of BDNF (*p* = 0.09).

### Behaviorally Stimulated Hippocampal ACh Efflux Is Blunted by PTD-Treatment, Which Can Be Selectively Restored by Exercise, but Is Dependent Upon the Actions of NGF

Overall, PTD treatment blunted behaviorally stimulated hippocampal ACh efflux [Treatment × Phase interaction: *F*(2,242) = 11.095, *p* = 0.0001] and exercise ameliorated this deficit [see Figures 6B,D; Exercise × Phase interaction: *F*(2,242) = 3.63, *p* = 0.028]. Overall, the there was a main effect of microbeads on ACh efflux [*F*(2,121) = 16.753, *p* = 0.0001] and a Microbeads × Phase interaction on ACh efflux [*F*(4,242) = 15.572, *p* = 0.0001]. Specifically, blocking NGF appeared to be critical to ACh efflux, as Scheffe’s *post hoc* analysis revealed that rats with TrkA-IgG coated microbeads had an attenuated effect of ACh efflux during spontaneous alternation testing, compared with rats treated with PBS-coated microbeads (*p* = 0.001). Furthermore, Scheffe’s *post hoc* analyses revealed that in PTD-treated rats, the exercise-induced rise in ACh efflux was abolished with the application of TrkA-IgG coated microbeads (*p* < 0.01). In PF rats (Figures 6A,C), despite no significant effect of exercise on hippocampal ACh efflux (all *p*’s > 0.06), Scheffe’s test revealed that the TrkA-IgG coated microbeads reduced the ACh efflux (*p*’s < 0.05). In contrast, sequestering of BDNF, by TrkB-IgG coated microbeads, did not influence ACh efflux in PF rats (*p* = 0.16) or PTD rats (*p* = 0.21).

### Exercise Rescues the PTD-Induced Loss of the Nestin+/ChAT+ Phenotype, and NGF Is a Critical Mediator of This Cellular Recovery

The analyses from the overall population of cholinergic neurons revealed that PTD-treated rats had a decreased expression of cholinergic markers compared with PF-treated rats [*F*(1,121) = 14.621, *p* = 0.001; see Table 1A] and that exercise resulted in a higher number of cholinergic markers being expressed, compared with sedentary rats [*F*(1,121) = 46.639, *p* = 0.0001]. *Post hoc* analyses indicated that in PTD-treated rats, exercise led to a higher expression of ChAT neurons, compared to what was observed in stationary rats [*F*(1,60) = 34.843, *p* = 0.0001]. Interestingly, the effect of exercise to increase the number of neurons that expressed ChAT, relative to stationary wheel conditions, was also true for PF rats [*F*(1,67) = 13.675, *p* = 0.001].

**Table 1:** **(A)** Unbiased estimations of the number of cells/mm^2^ for total ChAT neurons, Nestin+/ChAT- neurons, ChAT+/Nestin+ neurons, within the MS/DB.

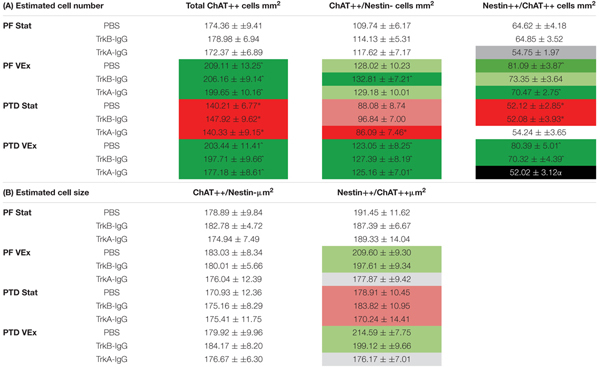

*PTD treated decreased the number of cholinergic neurons regardless of phenotype. Exercise increased both populations of cholinergic neurons. ***(B)*** Soma size of Nestin+/ChAT- and ChAT/Nestin+ neurons within the MS/DB. Overall, exercise increased the Nestin+/ChAT+ soma size in both PF and PTD rats, whereas TrkA-IgG reduced the soma size. Pink represents an overall main effect of Treatment; Red^∗^ indicates a significant *post hoc* effect of Treatment; Light green represents a main effect of Exercise, ˆ with dark green shading indicates a significant effect of Exercise within treatment conditions; Gray represent a main effect of microbeads, α with a black background indicates a significant effect of blocking NGF.*

To understand the extent of neuronal remodeling in the basal forebrain that occurs with exercise, we assessed the profile counts for the two cholinergic phenotypes: Nestin+/ChAT+ and ChAT+/Nestin-. There were a negligible number of Nestin+ cells that did not co-localize with ChAT (Nestin+/ChAT-; < 3 cells/mm^2^); and these non-co-localized cells did not vary in response to any manipulation, PTD treatment, exercise or microbead delivery (all *p*’s > 0.20). Importantly, the co-localization rates in our saline-treated rats did not differ from our previous measures employing confocal microscopy (see Hall and Savage, 2016). The overall ANOVA indicated that PTD treatment reduces the number of neurons expressing the Nestin+/ChAT+ phenotype [*F*(1,121) = 11.499, *p* = 0.001]. Exercise increased the expression of the Nestin+/ChAT+ phenotype [*F*(1,121) = 36.086, *p* = 0.0001], and this was evident in both PF [*F*(1,61) = 19.93, *p* = 0.001] and PTD-treated rats [*F*(1,60) = 16.65, *p* = 0.001]. Importantly, the Nestin+/ChAT+ neurons were sensitive to the sequestering of NGF during exercise exposure. There was main effect of microbead treatment [*F*(2,121) = 7.275, *p* = 0.001] and an Exercise × Microbead treatment interaction [*F*(2,121) = 3.29, *p* = 0.041]. Follow-up analyses, using Scheffe’s *post hoc* test, revealed that sequestering the actions of NGF inhibited the exercise-induced re-emergence of Nestin+/ChAT+ phenotype (*p* = 0.0001), while blocking BDNF had no effect (*p* = 0.100). Specifically, in PTD-treated rats there was a main effect of Exercise [*F*(1,60) = 16.651, *p* = 0.001] and a Exercise × Microbead interaction [*F*(2,60) = 5.354, *p* = 0.007], such that PTD VEx rats had a higher number of neurons expressing Nestin+/ChAT+, compared to PTD Stat rats (*p* = 0.005). In contrast, sequestration of BDNF did not block the effect of exercise to recover the Nestin+/ChAT+ phenotype in PTD rats (*p* = 0.104). In PF control rats, there was a main effect of microbeads [*F*(2,61) = 3.384, *p* = 0.04] and Scheffe’s *post hoc* test indicated that there was a trend for TrkA-IgG coated microbeads to decrease the Nestin+/ChAT+ expression in the stationary condition (*p* = 0.055). However, TrkB-IgG coated microbeads did not alter the Nestin+/ChAT+ neuronal expression in PF rats (*p* = 0.519).

Overall, inhibiting the actions of the neurotrophins was exclusive to the Nestin+/ChAT+ expressing neurons, since for ChAT+/Nestin- neurons there was no main effect of microbead application [*F*(2,121) = 0.552, *p* = 0.577]. However, for ChAT+/Nestin- neurons, there was an effect of exercise [*F*(1,121) = 29.649, *p* = 0.0001], such that exercise led to an increase in the number of ChAT+/Nestin- neurons, and this effect was seen in both PF [*F*(1,61) = 6.302, *p* = 0.015] and PTD rats [*F*(1,60) = 26.176, *p* = 0.0001].

### Exercise-Induced Hypertrophy of the Nestin+/ChAT+ Phenotype Is Abolished With Blockade of NGF

We assessed somatic area as this is mediated by NGF through the tyrosine kinase A receptor (TrkA-R; van der Zee and Hagg, 2002; Huh et al., 2008). Similar to our previously published data, PTD treatment had no effect on the somatic area on cholinergic neurons that did not express nestin [*F*(1,121) = 0.192, *p* > 0.66; see Table 1B]. Furthermore, neither exercise [*F*(1,121) = 0.503, *p* > 0.47], microbeads [*F*(2,121) = 0.303, *p* > 0.73], nor the interaction of these variables, altered the size of ChAT+/Nestin- neurons. In contrast, exercise increased the somatic area in the Nestin+/ChAT+ neurons [*F*(1,221) = 4.094, *p* = 0.04.5]. Importantly, there was a significant main effect of microbead treatment [*F*(2,121) = 3.338, *p* = 0.039], with *post hoc* analyses indicating that the blockade of NGF abolished the hypertrophy of the cholinergic neurons that expressed nestin (*p* = 0.02), but blockade of BDNF did not affect soma size (*p* = 0.48).

## Discussion

Exercise is neuroprotective and can lead to behavioral recovery following brain damage. The current data revealed that in the pathological brain, with cholinergic phenotype reduction, exercise revives the distinct subset of cholinergic neurons that co-express nestin. This exercise-induced cellular change is associated with a recovery of activity-dependent hippocampal ACh efflux and improved spatial memory performance. Furthermore, if exercise-induced changes in NGF are inhibited, the cellular and functional recovery of the septohippocampal cholinergic system is blocked. In contrast, exercise-induced changes in BDNF appear not to be critical for septohippocampal remodeling, as inhibiting the actions of BDNF does not obstruct exercise-induced recovery of cholinergic neurons, hippocampal ACh efflux, or spatial behavior.

However, in the intact brain, inhibiting the actions of BDNF does lead to a small, but significant, suppression of spatial behavior, regardless of exercise status. However, this deficit was not mirrored by changes in hippocampal ACh efflux or altered expression of cholinergic phenotypes. Baseline ACh levels were decreased in intact PF rats only with TrkA-IgG coated microspheres, further emphasizing that NGF is critical in modulating septohippocampal cholinergic tone, whereas BDNF may modulates behavioral outcome in intact animals through a non-cholinergic mechanism, such as effective synaptic plasticity or neurogenesis (see Vivar and van Praag, 2017). Furthermore, blocking the actions NGF in the intact brain impaired spatial behavior and suppressed ACh efflux, and without exercise, reduced expression of the cholinergic/nestin phenotype. It is well known that even in the intact brain that NGF, through activation of the Trk-A receptor, activates the cholinergic gene locus (Berse et al., 1999), to influence ChAT enzyme activity, vesicular ACh transporter (Berse et al., 1999), and enhanced high affinity choline uptake (Williams and Rylett, 1990), which modulate the ability of the neuron to express the cholinergic phenotype and release ACh. Interestingly, following exercise, the inhibition of NGF’s actions no longer suppressed the cholinergic/nestin phenotype.

Although there have been studies which demonstrated that exercise influenced the septohippocampal cholinergic system (Fordyce and Farrar, 1991; Ang et al., 2006; Hall and Savage, 2016; Vivar et al., 2016), none revealed which biochemical features of exercise modified the system. This series of studies revealed that NGF is a critical modulator of exercise-induced changes in the cholinergic forebrain system, in particular under pathological conditions. This matches the extensive literature that NGF has the capacity to rescue degenerating cholinergic neurons and drive remodeling of the septohippocampal circuit. Cholinergic neuronal identity (ChAT+), survival and cell size is regulated by NGF (Tuszynski and Gage, 1995; Naumann et al., 1997; Niewiadomska et al., 2002). Our data suggest that phenotypic plasticity of the cholinergic/nestin phenotype is also modulated by NGF.

### Loss of Cholinergic Neurons, Cognitive Dysfunction, and Recovery

Cholinergic cell loss is often not to be the sole pathology prompting cognitive impairment in neurodegenerative disorders, but it is still a key-inciting factor that contributes to behavioral dysfunction (Douchamps and Mathis, 2017). This is the scenario for the alcohol-related amnestic disorder caused by thiamine deficiency: The primary damage is thalamic cell loss (Mair, 1994; Savage et al., 2011), but we have demonstrated that exercise does not recover thalamic loss in the PTD rat model (Hall and Savage, 2016). Exercise produced robust improvements in spatial behavior and hippocampal ACh efflux, which were related to a recovery of the MS/dB cholinergic/nestin phenotype in amnestic PTD rats. These results further demonstrate that the loss of the forebrain cholinergic phenotype directly impacts learning and memory success, even when it is secondary pathology. Future studies need to demonstrate that direct NGF application into the hippocampus alone could fully recover the cholinergic phenotypes and recover the associated activity-dependent ACh efflux in amnestic rats.

After a neurotoxic event, cholinergic neurons enter an atrophic quiescent state in which they do not express the enzymes to maintain the cholinergic phenotype or transmission (Hagg et al., 1988; Tuszynski and Gage, 1995; Nagahara et al., 2009). However, a significant portion of cholinergic neurons (30–40%) can be rescued from this pathological state with timely and repeated exposure to NGF (Naumann et al., 1997; Gustilo et al., 1999). Cognitive recovery after NGF exposure requires 2–3 weeks (Markowska et al., 1996; Frick et al., 1997), which suggests the several behavioral effects of NGF are mediated by structural changes in cholinergic neurons. We previously found that recovery of the ChAT/nestin phenotype following 2-weeks of exercise required an additional 2-week restoration period (Hall and Savage, 2016). Nestin participates in the dynamic remodeling of cells throughout development. The expression of nestin in neural progenitor cells is regulated by other growth factors (EGF and FGF2; Pollard et al., 2006; Sun et al., 2008). Such findings support that idea that growth factors positively regulate nestin expression within some cells, and nestin modulates cell survival, which may or may not involves nestin’s critical role in the cytoskeleton integrity (Park et al., 2010). In the adult nervous system nestin appears to exert a cytoprotective function (Huang et al., 2009).

In 2002, Nestin+ cells were discovered in the MS/dB (Gu et al., 2002) that selectively co-localized with ChAT neurons, as no co-localization occurs in glia or other neuronal populations (GABAergic or glutamatergic; Wang et al., 2006; Guo et al., 2010; Hendrickson et al., 2011). Cholinergic/nestin neurons make up 35–45% of the total cholinergic neuronal population in the MS/dB in both humans and rats (Hendrickson et al., 2011), and these are mature neurons that have a higher excitability and received stronger spontaneous excitatory synaptic inputs than ChAT neurons that do not express nestin (Zhu et al., 2011). Finally, although ChAT/nestin neurons, relative to ChAT neurons without nestin, initially show greater sensitivity to colchicine-induced neurotoxicity, a larger proportion of ChAT/nestin neurons eventually recover (2–4 weeks) from neurotoxicity (Yu et al., 2011).

Although the precise role of nestin within ChAT neurons is unknown, the limited data suggest that nestin neurons within the basal forebrain are sensitive to aging and neuro-degeneration (Li et al., 2008; Hall and Savage, 2016; Fernandez and Savage, 2017). Li et al. (2008) have shown that in rats, Nestin+ neurons within the MS/dB decline with age, and that rat that displayed age-related memory impairment on Morris water maze had the lowest number of Nestin+ neurons. Furthermore, Nestin+ neurons in aged rats show reduced complexity with respect to dendritic arborization and dendritic length (Li et al., 2008). Such data support our hypothesis that the Nestin+ cholinergic phenotype is the more responsive cell population to neurotrophins.

The co-localization of nestin with ChAT might serve a neural recovery function that is mediated through the NGF-Ras-ERK signaling pathway (Huang et al., 2009). Our data suggest that a key difference between Nestin+ and Nestin- cholinergic neurons is a NGF-regulated type of plasticity that influences cholinergic marker proteins, cell shrinkage, neuronal survival, as well as synaptic efficiency, which ultimately influences attention, learning and memory performance.

### Different Roles of BDNF and NGF in Modulating Neuronal Plasticity, Learning, and Memory

Although there is evidence that BDNF and NGF have some overlapping trophic functions (see Castrén and Antila, 2017), their actions and timing within the septohippocampal circuit are different. General actions of BDNF occur on a constrained time scale (minutes to hours) and are activity-dependent at local synapses (Mcallister et al., 1996). For example, BDNF influences the induction of hippocampal LTP (Messaoudi et al., 2002), as well as transform early-phase LTP into late-phase LTP (Lu et al., 2008; Lynch et al., 2008). We recently demonstrated that BDNF rescues dysfunctional hippocampal LTP in the PTD-amnestic model (Vedder and Savage, 2017). The early effects of BDNF result from phosphorylation of synaptic proteins, whereas later effects mainly arise from transcriptional changes at the synapse (Leal et al., 2014). However, in the hippocampus, as well as other brain regions, changes in BDNF can persist through altered transcriptional control (Bramham and Messaoudi, 2005). Furthermore, blocking BDNF action with TrkB-IgG abolishes exercise-induced improvements in spatial learning (Vaynman et al., 2004). Although the TrkB receptor has high affinity for several neurotrophins, a more selective inhibition of BDNF signaling, via use of a BDNF siRNA, prevents enhancements of spatial learning seen following exercise (Intlekofer et al., 2013). Blocking the actions of acute BDNF via a TrkB antagonist or antibodies does impair the development of late LTP (Kang et al., 1997; Korte et al., 1998) and hinder consolidation and reconsolidation of inhibitory avoidance memory (Blank et al., 2016). It addition, acute blocking of TrkB receptors also blocks exercise-induced improvements in spatial memory (Korol et al., 2013). Thus, BDNF’s action on hippocampal function appears to be activity dependent. Although TrkB is also the receptor for NT-3 and NT-4 (Chung et al., 2013), blocking the TrkB receptor did not influence cholinergic recovery, thus the actions of NT-3 and NT-4 are also not likely to influence cholinergic recovery. Rather, TrkA-IgG was the important modulator, suggesting NGF is critical for the exercise-induced recovery in medial septal cholinergic function and release.

In contrast, although NGF is not capable of inducing hippocampal LTP, having NGF at the synapse can facilitate LTP (Kelly et al., 1998; Conner et al., 2009). Indeed, NGF is retrogradely transported from the hippocampus to the MS/dB, where it activates a series of transcriptional events that maintains the cholinergic phenotype (see Alderson et al., 1996), as well as the cholinergic tone within the septohippocampal circuit (for review, see Johnson et al., 1987; Mufson et al., 1999; Ito and Enomoto, 2016). While TrkA has been thought to be important for survival and differentiation, some cholinergic neurons also express the p75 receptor, which has been linked to cholinergic neuronal degeneration, and has an atrophic role in cholinergic basal forebrain neurons (Boskovic et al., 2014). This may suggest that p75 receptor expression is likely altered between nestin-expressing and non-expressing cholinergic neurons, as perhaps receptor distribution contributes to the exercise-induced cholinergic recovery. In a previous study (Hall and Savage, 2016), we found that exercise-induced enhancements of the cholinergic/nestin phenotype took weeks to emerge. In the degenerating human brain, viral delivery of NGF slowly promoted axonal sprouting and hypertrophy in cholinergic neurons that persists across months (Tuszynski et al., 2015). Thus, it appears that NGF regulates the effectiveness of the forebrain cholinergic system on a protracted time frame (Conner et al., 2009). Although BDNF can also improve cholinergic function and structure, it is not as effective at doing so as NGF (Morse et al., 1993). Our data revealed that exercise-induced increases in BDNF are not involved in the boosting of cholinergic structure or function within the septohippocampal circuit.

## Conclusion

We revealed an association between the exercise-related improvement of spatial memory and the improvement of septohippocampal cholinergic system. Augmentation of neurotrophin levels is a key feature of exercise that leads to improved cognitive outcomes. We determined that exercise-induced changes in NGF are critical for restoring waning cholinergic/nestin neurons, blunted hippocampal ACh efflux and impaired spatial behavior following thiamine deficiency. Furthermore, in the intact brain inhibiting the actions of NGF reduces the effectiveness of exercise on behavior and hippocampal ACh efflux, without changes in the cholinergic/nestin phenotype. Interestingly, exercise-induced changes in BDNF are not critical for restoring/elevating septohippocampal function. However, our data demonstrate an important avenue (beyond neurogenesis) by which exercise changes cognitive outcome: modulation of the cholinergic system, particularly in the pathological brain. Given that cholinergic function is the most documented neural substrate of cognition, and that cholinergic dysfunction is commons in an array of neurodegenerative diseases (Schliebs and Arendt, 2011), exercise should be considered as a therapeutic for disorders of cholinergic function.

## Author Contributions

JH and LS designed and planned the study, analyzed and interpreted the results, and contributed toward the final version of the manuscript. FG-P assisted in developing the manuscript. JH carried out the experiment with support and assistance from FG-P.

## Conflict of Interest Statement

The authors declare that the research was conducted in the absence of any commercial or financial relationships that could be construed as a potential conflict of interest.
